# ZERO: Probabilistic Routing for Deploy and Forget Wireless Sensor Networks

**DOI:** 10.3390/s101008920

**Published:** 2010-09-29

**Authors:** Xavier Vilajosana, Jordi Llosa, Jose Carlos Pacho, Ignasi Vilajosana, Angel A. Juan, Jose Lopez Vicario, Antoni Morell

**Affiliations:** 1 Universitat Oberta de Catalunya, Rambla Poblenou 156 08018 Barcelona, Spain; E-Mails: jllosa@uoc.edu (J.L.); ajuanp@uoc.edu (A.A.); 2 Universitata Politecnica de Catalunya, Jordi Girona, 08034 Barcelona, Spain; E-Mails: jcpacho@est.fib.upc.edu (J.C.P.); ignasi.vilajosana@upc.edu (I.V.); 3 Universitat Autonoma de Barcelona, 08193 Bellaterra (Barcelona), Spain; E-Mails: jose.vicario@uab.es (J.L.V.); antoni.morell@uab.cat (A.M.)

**Keywords:** energy efficiency, probabilistic routing, collection-tree-protocol, gradient routing, wireless sensor networks, agriculture and industry

## Abstract

As Wireless Sensor Networks are being adopted by industry and agriculture for large-scale and unattended deployments, the need for reliable and energy-conservative protocols become critical. Physical and Link layer efforts for energy conservation are not mostly considered by routing protocols that put their efforts on maintaining reliability and throughput. Gradient-based routing protocols route data through most reliable links aiming to ensure 99% packet delivery. However, they suffer from the so-called ”hot spot” problem. Most reliable routes waste their energy fast, thus partitioning the network and reducing the area monitored. To cope with this ”hot spot” problem we propose ZERO a combined approach at Network and Link layers to increase network lifespan while conserving reliability levels by means of probabilistic load balancing techniques.

## 1. Introduction

As Wireless Sensor Networks (WSN) are being adopted by industry and agriculture and their use is being widespread out of academical environments the need for long lifespan (The lifetime of a long-lived WSN is typically expected to be on the order of years with node duty cycles less than 1%. e.g., less than 1 sample per minute), robustness and throughput are becoming critical points for the success of any application. Industry addresses real scenarios usually with well defined requirements and constraints which do not always fit with the research carried out at the university. Whilst numerous protocols and techniques have been developed to ensure energy efficiency, 99.9% delivery ratio and efficient routing protocols, more work is still needed to provide a robust implementation to attain the deploy and forget wireless sensor networks. Due to the heterogeneity of industrial scenarios and possible applications of WSN, the combination of techniques does not always provide a good candidate solution. In this paper we address those scenarios where WSN have to be deployed to collect data for very long periods of time and, therefore, information lost becomes critical (e.g., natural hazard monitoring, smart city applications, metering, *etc.*). We aim to implement an energy efficient routing protocol combined with an improvement of the reliability of data transmission whilst keeping a balanced energy consumption with respect to delivery ratio. Besides a key objective is to minimize protocol configuration and its dependency to the addressed scenario.

Energy consumption is the key limitation of long lived WSN which is strictly dependant to transmission rates. Gradient routing protocols [1] usually overload best paths to the sink, *i.e.*, nodes with particularly favourable links are thus likely to have a heavier workload than their neighbours as they are chosen to relay traffic that they do not generate. The additional overload reduces the lifespan of these critical nodes and leads to network partitioning [2], usually known as hot spot problem. For wide area phenomena monitoring, path elimination (aka the hot spot problem) not only diminishes the reliability of the network but also reduces the phenomena monitoring which becomes in a unacceptable behaviour. Therefore, this paper presents our advances to customize routing and reliability of deploy and forget WSN considering the trade-off between reliability and energy consumption. On the one hand, at Network layer a generalization of the Collection Tree Protocol (CTP)[3] is presented which enables probabilistic load balancing of routes to the sink. Due to its randomized nature, the proposed protocol is autonomous and decentralized only requiring each node to probabilistically decide the path to the sink. On the other hand, at the Link layer, retransmission for reliability is also introduced. The paper evaluates the combination of both techniques in a real deployment.

## 2. Related Work

Despite numerous research efforts [4–9], WSN routing remains to this day a fairly open issue. In this section we will only focus on mote-oriented routing protocols that have been implemented on actual sensor network platforms. A number of cost-based routing protocols have been developed for motes using TinyOS [10]. MintRoute [1], MultiHopLQI [11], Collection Tree Protocol (CTP) [3], Routing Protocol for Low power and Lossy Networks (RPL) [12] and Arbutus [13] represent successive evolutions of a common cost-based paradigm defined in [1]. Link estimation is seen as an essential tool for the computation of reliability-oriented route selection metrics. Basically those routing protocols address scenarios characterized by one sink (excepting CTP and Arbutus) and where nodes generate traffic periodically at a fixed rate. Routing protocols use gradient metrics to determine next hop while keeping some traffic in the network to maintain information about link quality. Routing of packets then is based on the selection of the best candidate next hop. MintRoute makes use of routing tables that keep the Expected Number of Transmissions (ETX) metric [14] of all of the neighbours of a node. In order to prevent tables from growing indefinitely, it makes use of a neighbourhood management policy [15].

MultiHopLQI, does not use routing tables. A new parent in the routing tree is adopted if it advertises a lower cost than the current parent (*i.e.*, next hop candidate node). The link metric is Link Quality Information (LQI) which is used additively to obtain the gradient of a given route. MultiHopLQI only keeps state for the best parent at a given time which drastically reduces memory usage and control overhead. CTP, uses ETX as link estimation. Routes are selected by according to the minimum gradient (*i.e.*, sum of the costs of the route) and transmission deferrals are used in case of parent node congestion. The key feature of CTP is that it adjust control and state maintenance beaconing by means of the Trickle algorithm [16]. RPL improves CTP by enabling downstream communication (from the sink to the nodes). To the best of our knowledge, Arbutus is the unique protocol that pursuits load balancing. As MultiHopLQI, it does not keep routing tables but only the best candidate next hop for each node is kept. It builds a tree topology and beaconing is only carried out in a top down approach. Next hop estimation is calculated taking into account bottleneck information (which provide inherent load-balancing) and Channel State Information, LQI in IEEE802.15.4 protocol [17]. As authors outline in their work, even Arbutus performs as well as MultiHopLQI with a 30% energy savings, the calibration of the protocol is not trivial and depends of every scenario. Finally, our work pursuits the same objectives as Arbutus, but also simplifying the fine-tuning process of the network.

## 3. Generalization of CTP

### 3.1. The Collection Tree Protocol (CTP)

CTP is a tree-based collection protocol intended to provide best effort routing. In the CTP some number of nodes advertise themselves as tree roots of the network. Then other nodes in the network form a set of routing trees to these roots. A particularity of CTP is that the network is address-free (*i.e.*, a node does not send a packet to a particular root). Instead, nodes implicitly choose a root by choosing a next hop, best routes to the sink node are determined by using a gradient metric. CTP uses expected transmissions (ETX) as its routing gradient. A root has an ETX of 0. The ETX of a node is the ETX of its parent node plus the ETX of its link to its parent node. Given a choice of valid routes, CTP chooses the one with the lowest ETX value. A perfect link has an ETX of 1, however, certain implementations such as the CTP implementation for TinyOS uses the value of 10.

Routing loops are a problem that can emerge in a CTP network. Routing loops generally occur when a node choose a new route that has a significantly higher ETX than its old one, perhaps in response to losing connectivity with a candidate parent. If the new route includes a node which was a descendant, then a loop occurs.

CTP addresses loops through two mechanisms. First, every CTP packet contains a node’s current gradient value. If CTP receives a data frame with a gradient value lower than its own, then this indicates that there is an inconsistency in the tree. CTP tries to resolve the inconsistency by broadcasting a beacon frame, with the hope that the node which sent the data frame will hear it and adjust its routes accordingly. If a collection of nodes is separated from the rest of the network, then they will form a loop whose ETX increases forever. CTP’s second mechanism is to not consider routes with an ETX higher than a reasonable constant. The value of this constant is implementation dependent.

## 4. CTP and Probabilistic Routing

As stated previously, CTP suffers from the hot spot problem. Best routes are rapidly overloaded leading to fast energy depletion and network partitioning. This problem can be diminished by several load balancing schemes to route information by other well connected routes. One way to address load balancing is to introduce new parameters to be measured by the control plane (state information transmitted to keep the topology of the routing protocol). These techniques introduce new traffic to the network and usually require blacklisting techniques to avoid bottleneck nodes. Blacklisting usually require application specific configuration in order to avoid network partitioning [18].

In order to reduce the hot spot problem, we propose to use probabilistic techniques to determine routes. The idea behind our work is to select a route based on probabilities. Eligibly routes will be selected taking into account CTP routing metric and the neighbour table information of a node. Probabilities will be assigned to possible paths considering local information of each node. In order to avoid pure randomized choice (as Random Walks [19]), a biased selection pattern is introduced. In our work two metrics have been studied, the first one calculates distances between possible paths and assigns a probability proportional to the quality of that path with respect to others. The second technique uses a probability distribution function that assigns a probability to each possible path without requiring in-node calculations. Both techniques give more probability to the best route (*i.e.*, represented by less ETX) in order to do not heavily compromise the reliability of the network.

### 4.1. Proportional Distance Metric

Node routing tables keep information of the ETX to some of their neighbours, CTP protocol selects the route with minimum ETX as a candidate for transmission. In order to balance the number of packets transmitted through a route, we investigated the use of a proportional probabilistic metric based on the differences between ETX of candidate paths.

In order to determine the parent for the next transmission a subset Λ of *k* (where k ≥ 1) neighbours is selected. The subset is formed by the *k* neighbours with minimum ETX excluding the current parent. The selected neighbours and the current parent are then divided in two groups (group one, and group two). Those with ETX at a distance lower than a given threshold *θ* > 0 of the best candidate neighbour, and those ones with higher distance but not overpassing a threshold *ω*. Within those groups, nodes are assigned weights according to the following formula:

$$\forall_{i,j}\in k\;w_{i}=\{\begin{array}{ll} |\theta -i_{\mathrm{etx}}{|}^{\alpha } & \mathrm{if}\;i_{\mathrm{etx}}-\min(j_{\mathrm{etx}})<=\theta \\ |\omega -i_{\mathrm{etx}}{|}^{\alpha } & \mathrm{if}\;\theta <i_{\mathrm{etx}}-\min(j_{\mathrm{etx}})<=\omega \\ \infty & \mathrm{otherwise} \end{array}$$

Textually, the weight of a neighbour *i* depends on the distance to the best candidate node, (*i.e.*, the neighbour with minimum ETX). If this distance is lower than a *θ* weight is computed as the result of calculating the distance between *θ* and the ETX of neighbour *i*. In the other case, considering nodes in group 2, the weight is calculated using the threshold *ω*. This distinction is done to maximize distances between good candidates and not so good candidates. The *α* parameter is used to magnify differences.

A constraint in our algorithm is also introduced. Weights for nodes in group one constitute the σ% (98% in our implementation) of the weight of the nodes in the subset Λ This is motivated by the fact that with 1 − σ% (2% in our implementation) probability other paths out of the best ones eventually will be explored.

Once nodes have been assigned a weight, probabilities of being selected are assigned as a proportion of the quotient between their weight with respect to the total weight of the *k* neighbours in Λ, *i.e.*,

$$p_{i}=w_{i}/\sum w_{i}$$

Finally for each group a random number *δ* drawn from the uniform distribution in the interval (0, Σ(*w_i_*)) is chosen. A lottery scheme is used to chose the parent. *w_i_* represents the number of tickets of a candidate *i* and the *δ* represents the ticket. A neighbour *i* is elected if:

$$\sum (w_{i})\geq \delta$$

Considering that neighbours are sorted in ascending order of weight. Once there is a winner for both groups, another ticket is issued with probability σ for the first group and (1 − σ) for the second one.

The value of *θ* should be 10 ETX as 10 is the minimum ETX for one hop. This value ensures that any node within the group is not descendant of any other node in the group. For *ω* the suggested value is *ω* > 50 ETX. For CTP implementation a node is discarded as neighbour if its ETX falls 50 (MaxLinkQuality). Then *ω* > 50 ETX is selected in order to do not definitively discard the node since eventually it can become a good candidate.

### 4.2. Triangular Distribution Function

In order to model the distribution of the possible routes to the sink we investigated the use of a discretized version of the Triangular Distribution Function (TDF) as suggested in [20]. The Triangular Distribution is typically used as a subjective description of a population for which there is only limited sample data. It is based on a knowledge of the minimum and maximum and an inspired guess as to what the modal value might be.

$$f(x|a,b,c)=\{\begin{array}{ll} \frac{2(x-a)}{\left(b-a)(c-a\right)} & \text{for}\;a\leq x\leq c\\ \frac{2(b-x)}{\left(b-a)(b-c\right)} & \text{for}\;c\leq x\leq b\\ 0 & \text{otherwise} \end{array}$$

*a*, *b* and *c* parameters determine the form of the probability density function. Parameter *a* represents the lower limit, *c* represents the mode and *b* the upper limit. For basic CTP the distribution function uses *a* = *b* = *c* with the value of 1 indicating that there is only one possible neighbour eligible. When multiple paths to the sink are considered, the *b* parameter indicates the number of candidate neighbours eligible for transmission. Having characterized the Probability Density Function it is quite simple to generate random variates that follow the distribution.

**Figure 1.**
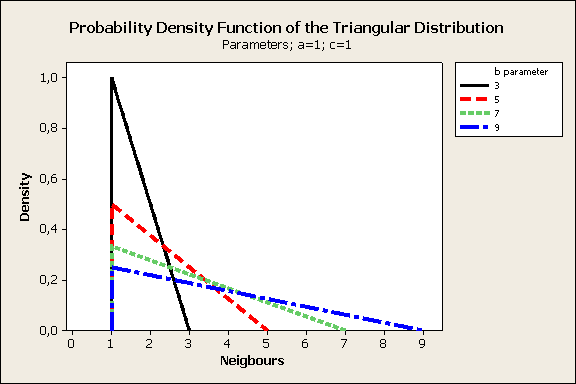
Plot of different probability density function of the Triangular Distribution. The X axis indicate the number of eligible neighbours.

**Figure 2.**
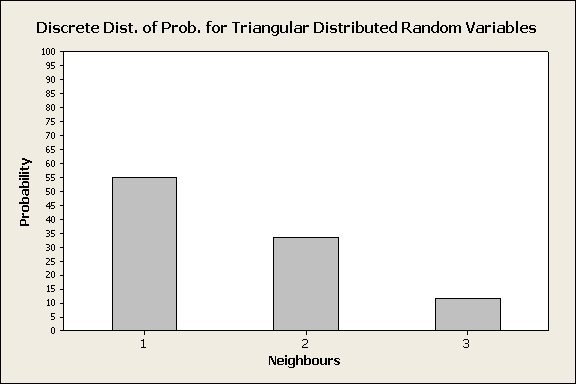
Discrete Probability Function for Triangular Distribution. For 3 neighbours the probabilities are distributed following the Probability Density Function of the TD.

In order to determine the candidate node for transmission, instead of selecting the node with minimum ETX as CTP does, nodes are logically sorted by ascending ETX. The node with lowest ETX will be considered the first candidate, and other nodes will be considered subsequent candidates according to their ETX. The neighbours to transmit are selected according to the Probability Distribution Function of the Triangular Distribution (see Figures 1 and 2).

Before each transmission each node computes a value *μ* drawn from the uniform distribution in the interval (0, 1). The neighbour to transmit to is calculated as the floor of *X*, a triangular distributed random variate given by:

$$X=b\times (1-\sqrt{1-\mu })$$

This schema distributes transmissions amongst candidate paths but giving more chances to transmit to well connected nodes.

## 5. Link layer Optimization

In order to increase the energy efficiency of the communication protocol several improvements to the BMAC [21] have been introduced. A receiver oriented slotted MAC have been implemented. Under this model time is divided into frames of a fixed duration. Each node chooses a slot within a frame and listens only on its designated slot for a short duration (in our implementation 6ms–15ms depending on the payload length). Other nodes willing to send to this node have to synchronize and send during its corresponding slot.

Likewise the Low Power Listening (LPL) when a node aims to send for the first time to any other node, it sends for the duration of the overall frame in order to synchronize with a receiver. Any of the possible receivers has a slot scheduled within this period, therefore, upon reception of the packet, the receptor will reply an acknowledgement message (ACK). The transmitter receives the ACK and thus knows the moment when that particular node has its slot scheduled. Further transmission will wait until the receiver’s slot to transmit and update the receptor schedule to compensate for the clock drift.

During a transmission, since transmitter could have been desynchronized, the transmitter sends continuously for the duration of a window. If a send fails, the transmitter keeps a history of NACKs for each neighbour. The duration of the send window is proportional to the number of NACK for that particular neighbour. Broadcast packets contrarily are sent for the duration of the whole frame.

The wake schedule is implemented by a periodic timer. A node powers up the radio at each period and listens for a period of time determined by their listen period that should not be shorter than the double of the round trip time.

$$T=2\times (T_{\mathrm{ttime}}+T_{\mathrm{ack}})$$

Neither a scheduled listening slot nor a sending request will ever interrupt other ongoing operations. If a send is taking place and a scheduled listening slot fires, the listening slot is lost. If this happens too many times (50 in our implementation), a new slot that does not collision with neighbours’ slots is chosen.

Any node gathers information about all neighbours that the node has sent a message at some time. The neighbour table’s size is our implementation is 20. It is used to save information about the last successful transmission, the number of NACKs so far since the last successful transmission and the number of packets sent to that neighbour. If a new neighbour is discovered and the table is full, a bad neighbour (any of those with NACK≥5) is substituted by the new node.

### 5.1. Send Operation

The send operation have been divided in 3 phases. Let’s consider a node *A* aiming to send to a node *B*.

- The neighbour (*B*) is identified. If a *A* has a successful send to *B* within the last 3 ms, *A* starts sending immediately. Otherwise *A* calculates its next listening slot and sleeps until it happens. If previous sends to *B* have failed (*i.e.*, NACK>0), *A* does not select the next listening slot in order to send the packet, but one slot randomly chosen within the following 3 slots. Broadcasts and sends to unknown nodes are started immediately.
- Within the listening slot of *B*, *A* starts sending for a duration *D* where *D* = *NACK* * *window*. Broadcasts and sends to unknown nodes are scheduled to send for the whole frame. If *A* receives an ACK or the sending time runs out, whichever comes first, the sending is stopped. If an ACK is received, *A* updates the neighbour’s table information.
- After the send has been completed a node waits further 3 ms to see if any other send request has the same destination. If no other send request arrive the radio is turned off.

## 6. Experimentation

In order to evaluate ZERO a set of different studies have been carried out. Simulation and real implementation testing have been combined in the evaluation.

### 6.1. Simulation Experiments

CTP and ZERO have been implemented in JAVA in order to be able to evaluate them in a large scale. The experiment constructed a network as a k-ary tree with n nodes.

$$n=\frac{k^{h+1}-1}{k-1}$$

The maximum depth was set to five hops (h = 5), a maximum number of child nodes of six (k = 6) per each node and a unique sink which leads to a network of 9,331 nodes. Initial ETX rates have been assigned following a uniform distribution. The simulation aimed to evaluate the number of messages routed by each node in the network and compare the approaches taken by CTP and ZERO. In the experiment each node aimed to transmit 1,000 packets which were routed to the sink following the route determined by CTP or ZERO. For ZERO the triangular distribution metric with *a* = *c* = 1 and *b* = 3 was selected as route determination policy. The probability of successful transmission was set in the range of 80% to 99.9% since we aimed to evaluate the behaviour of the protocols under considerably low failure rates. Experiments were repeated 50 times and average results are presented.

**Figure 3.**
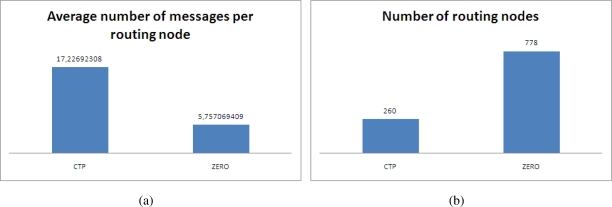
Number of messages per routing node and number of nodes routing information from other nodes. (a) Average number of messages per routing node (in average for the 50 repetitions of the experiment), (b) Number of nodes routing messages from other nodes.

Figure 3 compares the results obtained in our simulation regarding the number of packets relayed by each routing node (Node that routes information from other nodes) in CTP and ZERO. For the CTP implementation, routing nodes transmitted in average 17.23 messages whilst nodes in ZERO transmitted 3 times less messages (5.76 in average). This is attributed to the probabilistic distribution of routes in ZERO that in average balances the load in the *b* paths. However the distribution is not equitable for each of the path but best paths will keep transmitting more packets. Figure 3(b) shows the number of nodes routing information from other nodes.

**Figure 4.**
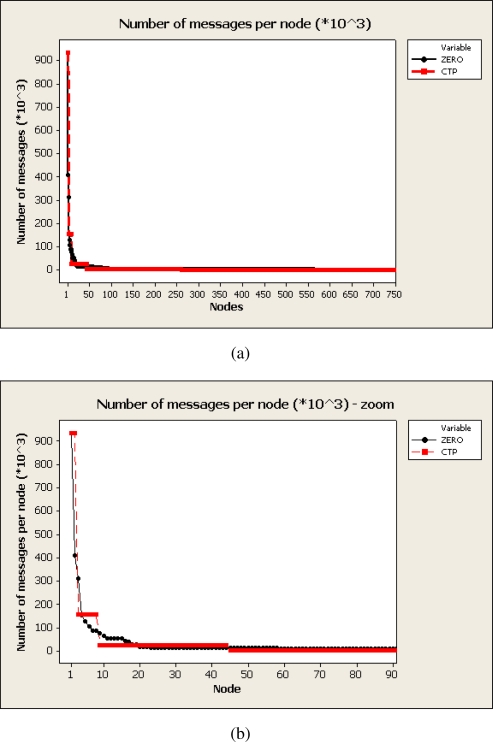
Number of messages routed per node. X-axis show nodes descending sorted by the number of messages routed. Node 1 represents the root node as it routes all messages generated in the network. (a) Distribution of number of messages routed per each node, (b) Zoom in of the previous image.

Figure 4 presents the number of messages relayed by nodes in the network. Nodes have been sorted in a decreasing way as regards to the number of packets transmited. Figure 4(a) shows only 778 out of the 9,331 nodes in the network since the rest of nodes did not relayed packets from others (only transmitted their 1,000 packets). Root nodes (node 1 in the figure) from CTP and ZERO almost transmitted the same number of messages, around 920,000 in average with slight variations attributed to the randomized probability of successful transmission attributed to each node. It can be seen either in CTP and ZERO that the distribution of transmitted packets follows a power law where few nodes have transmitted the majority of the information. As the overlay created by CTP and ZERO are trees this is the expected distribution. However, looking at Figure 4(b) it can be seen that for CTP the number of packets transmitted are distributed in a pseudo-step distribution. Relay nodes at the same depth in the network transmit almost the same number of messages due to the homogeneity of the network. For ZERO the number of transmitted packets are distributed more uniformly due to the fact that loads are triangular distributed and consequently two consecutive transmission does not always follow the same path even prioritizing best routes.

**Figure 5.**
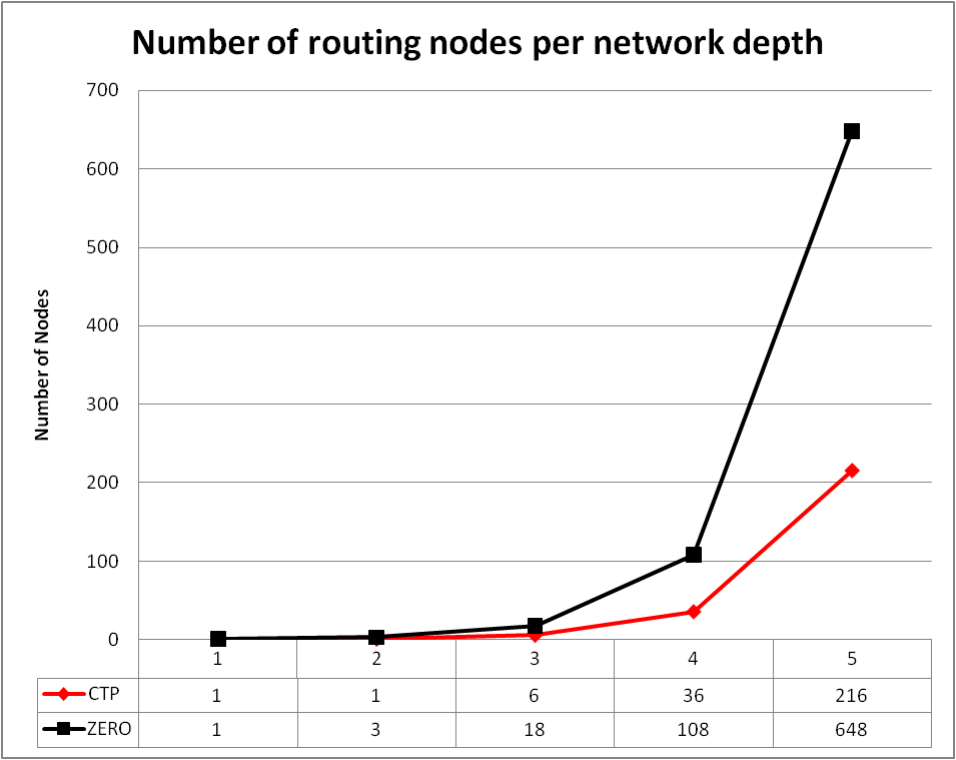
Average number of messages per relay (in average for the 50 repetitions of the experiment).

Figure 5 show in average the number of routing nodes at different depth of the network. Root nodes route all packets but at subsequent network depth the ratio of nodes routing information is 1:3 for CTP in respect to ZERO. The results obtained by the simulation demonstrated that ZERO balances the load at routing nodes by introducing new suitable paths for transmission to the sink reducing then the number of transmitted packets per node. As a consequence, energy depletion of hot spots is also mitigated.

### 6.2. Real Deployment Experiments

**Figure 6.**
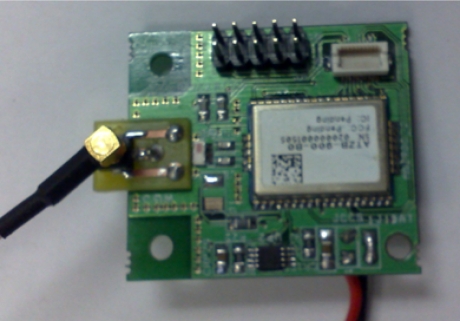
WorldSensing G3 mote used in the experimentation.

In a real evaluation we aimed to investigated whether ZERO compromises the reliability of the network. The experiments were conducted using the G3 node from WorldSensing (Figure 6). The G3 nodes are equiped with an Atmel Atmega microcontroller and a CC2420 radio at 866Mhz. They make use of TinyOS as operating system. Both triangular and proportional metrics of ZERO have been implemented. As CTP regards, the TinyOS implementation have been used. Finally the evaluation of both routing techniques has been carried out using our receiver oriented MAC layer.

A network of 10, 21 and 54 motes have been deployed at our lab. Motes were randomly placed indoor and outdoor with the restriction of ensuring full network connectivity. Experiments for 10 nodes were repeated 102 times, for 21 nodes 30 times and for 54 nodes 10 times for both CTP and ZERO routing protocols. In total data from 2197 nodes were collected. Delivery ratio as a proportion of packets issued w.r.t acknowledgements received was computed for each node. For ZERO this time the proportional distance metric has been used.

**Figure 7.**
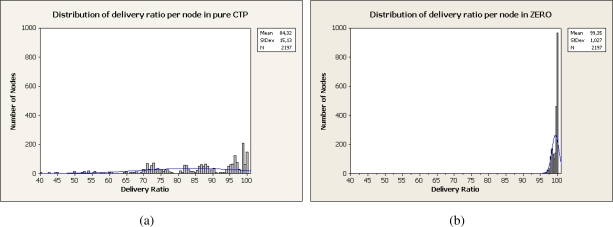
Distribution of delivery ratio per node. (a) CTP, (b) ZERO.

Figure 7 shows the distribution of the delivery ratio for all the studied nodes. For CTP implementation nodes exhibit a mean delivery ratio of 84.32% while for ZERO a 99.35%. That 15% of increment is attributed to the path diversity introduced by ZERO that reduces the probability of collision at a node. The effects of selecting other than the optimal path calculated by CTP are inappreciable in our experiments due to that they are compensated by the reduction of collisions at relaying nodes.

Regarding energy consumption a cost-benefit metric *η* has been used as in [13]. In our scenario nodes transmitted at the same rate in a many to one fashion so the only factor that introduced variations in the energy consumption of individual nodes depends on the delivery ratio of routing nodes to the sink (*i.e.*, the number of retransmission due to failure in the delivery or due to multiple hops). Likewise [13] the cost benefit function has been calculated as a ratio of the total number of packets received by the sink with respect to the total relayed packets by routing nodes. Let N be the total number of nodes in the network, let R be the subset of nodes routing information. The benefit function then is the total number of issued packets in the network w.r.t the cost function. The cost function calculates the total number of retransmissions of packets until they reach the sink.

$$\eta =\frac{\sum_{i\in N}P_{i}}{\sum_{j\in R}\mathrm{ET}X_{j}^{-1}}$$

**Figure 8.**
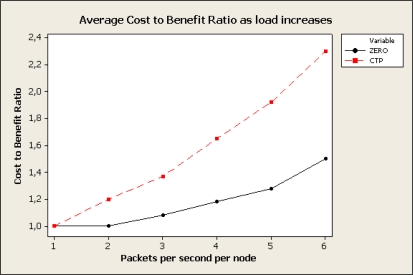
Cost benefit ratio for different loads of the network.

Figure 8 shows the cost-benefit ratio of CTP and ZERO as regards to the load of the network. Due to path diversity ZERO mitigates the effects of congestion, represented as rapid increase of the ETX in CTP due to unsuccessful packet transmissions. The experiment was carried out using 21 motes generating traffic to the sink. Experiment was repeated for different packet rates (1 to 6 packets per second). Although ZERO not always favours the most reliable link it outperforms CTP as congestion increases.

**Figure 9.**
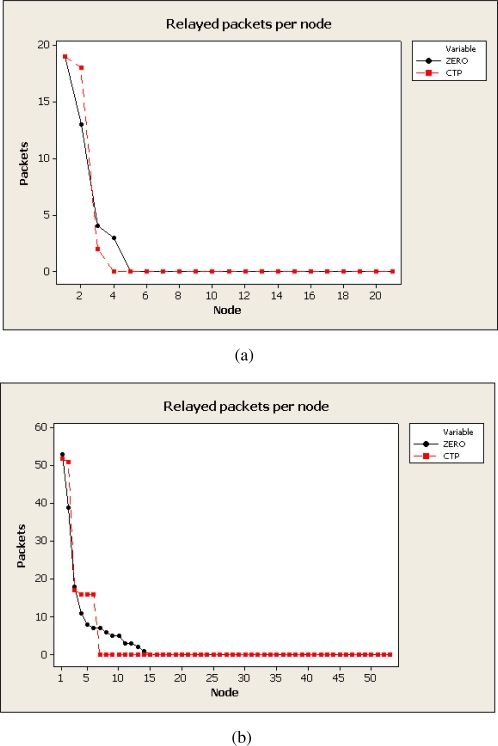
Number of packets relayed per node. (a) Relayed packets per node in the 21 nodes deployment, (b) Relayed packets per node in the 54 nodes deployment.

The Figure 9 presents the number of packets relayed by different nodes in the network. The data presented in Figure 9 has been obtained for the deployments of 21 and 54 motes. ZERO again makes use of more relay nodes but the load of all of them (excepting sink node) is lower than most overloaded nodes in CTP.

**Figure 10.**
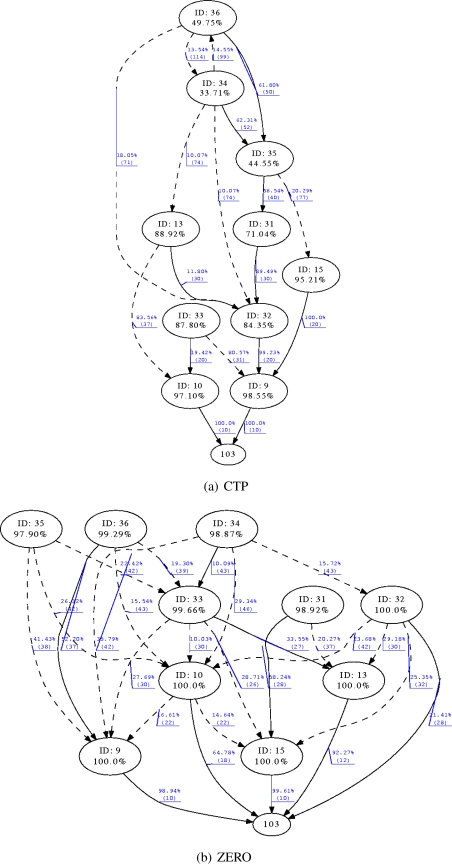
Routing topology created by CTP and ZERO for 11 motes and the same physical topology.

Finally Figure 10 shows the results of an experiment of 11 nodes for both CTP and ZERO. The diagrams present the topology of the network created by the routing protocol. Figures for experiments of 21 and 54 nodes have not been included due to their size and the confusing amount of information. Circles represent nodes identified by their ID and below the mean delivery ratio achieved during the experiment. Continuous edges represent most used path to the sink whilst dotted edges represent alternative path also used along the experiment. Each edge indicates the percentage of packets that traversed that path towards the sink whilst in brackets the ETX from the source of the edge to the sink is shown. Slight variations in ETX for both experiments can be attributed to the inherent dynamism of the wireless medium since both experiments have been carried out at different moments in time despite of using the same nodes and physical topology.

Figure 10(a) presents the routing tree created by CTP and Figure 10(b) the tree created by ZERO. Node with ID 103 acted as the sink of the network in both experiments. At a first glance, CTP built up a tree with a maximum hop count of 5 whilst ZERO constructed the tree with a maximum depth of 3. Nodes 9 and 10 acted as a last hop to the sink in both experiments, ZERO indeed allowed nodes 32, 13 and 15 to directly communicate to the sink which flattened the tree and reduced the overall number of hops. As ZERO probes alternative routes even having a major cost, the tree tends to flatten which eventually reduces the hop count and consequently the probability of delivery failure. For example node 36 exhibited a 49.75% of delivery ratio using CTP and a path to the sink of 5 hops and ETX of 50. In ZERO the same node achieved a delivery ratio of 99.29% in a 3 hops path to the sink and best ETX of 37.

## 7. Conclusions

The paper presented ZERO a probabilistic routing protocol designed as a generalization of the Collection Tree Protocol. ZERO load balances the traffic of the network by means of oriented but probabilistic path selection. Two metrics for path selection have been presented. A proportional distance metric assigns weights to candidate path as a proportion of their quality with respect to the quality of the possible paths. A triangular distribution based metric assigns path probability according to a triangular distribution function. Both techniques introduce randomization to path selection augmenting the number of paths to the sink. The choice for both techniques is biased to the best candidate in order to do not compromise the reliability of the overall network. A series of studies through simulation and real deployment have been performed. The objective was to validate the suitability of randomized path selection compared to restrictive path selection of CTP that suffers from the hot spot problem. Our studies showed that ZERO outperforms pure CTP in terms of delivery ratio as CTP under congestion suffers from high amount of packet collisions. Besides, the experiments showed that ZERO makes use of more relaying nodes but these nodes in average transmit much less packets, leading then to a more balanced energy consumption. The paper did not compare both metrics presented which we let as a future work, but however some directions have been already shown. The distance metric would require certain tuning depending on the network, but once tuned it would outperform triangular distribution metric which is very simple to implement and provides a very interesting load balancing.

## References

[1] Woo A, Tong T, Culler D Taming the underlying challenges of reliable multihop routing in sensor networks.

[2] Haenggi M (2003). Energy-Balancing Strategies for Wireless Sensor Networks. ISCAS.

[3] Gnawali O, Fonseca R, Jamieson K, Moss D, Levis P (2009). The Collection Tree Protocol (CTP).

[4] Chen JL, Ma YW, Lai CP, Hu CC, Huang YM (2009). Multi-Hop Routing Mechanism for Reliable Sensor Computing. Sensors.

[5] Lee JH, Jung IB (2010). Speedy Routing Recovery Protocol for Large Failure Tolerance in Wireless Sensor Networks. Sensors.

[6] Figueiredo CMS, Nakamura EF, Loureiro AAF (2009). A Hybrid Adaptive Routing Algorithm for Event-Driven Wireless Sensor Networks. Sensors.

[7] Kandris D, Tsioumas P, Tzes A, Nikolakopoulos G, Vergados DD (2009). Power Conservation through Energy Efficient Routing in Wireless Sensor Networks. Sensors.

[8] Joshi GP, Kim SW (2009). A Distributed Geo-Routing Algorithm for Wireless Sensor Networks. Sensors.

[9] Garca Villalba LJ, Sandoval Orozco AL, Trivio Cabrera A, Barenco Abbas CJ (2009). Routing Protocols in Wireless Sensor Networks. Sensors.

[10] Levis P, Madden S, Polastre J, Szewczyk R, Whitehouse K, Woo A, Gay D, Hill J, Welsh M, Brewer E, Culler D (2005). TinyOS: An Operating System for Sensor Networks.

[11] TinyOS. MultiHopLQI. Available online: http://www.tinyos.net/tinyos-1.x/tos/lib/MultiHopLQI/ (accessed on 20 September 2010).

[12] Tim Winter RT (2010). RPL: IPv6 Routing Protocol for Low Power and Lossy Networks.

[13] Puccinelli D, Haenggi M Arbutus: Network-Layer Load Balancing for Wireless Sensor Networks.

[14] De Couto DSJ, Aguayo D, Bicket J, Morris R (2005). A High-Throughput Path Metric for Multi-Hop Wireless Routing. Wirel. Netw.

[15] Demaine ED, López-Ortiz A, Munro JI (2002). Frequency Estimation of Internet Packet Streams with Limited Space.

[16] Levis P, Patel N, Culler D, Shenker S Trickle: A Self-Regulating Algorithm for Code Propagation and Maintenance in Wireless Sensor Networks.

[17] IEEE (2006). Wireless MAC and PHY specifications for low rate WPAN, IEEE Std 802.15.4-2006 (Revision of IEEE Std 802.15.4-2003).

[18] Gnawali O, Yarvis M, Heidemann J, Govindan R Interaction of Retransmission, Blacklisting, and Routing Metrics for Reliability in Sensor Network Routing.

[19] Mabrouki I, Lagrange X, Froc G (2007). Random Walk Based Routing Protocol for Wireless Sensor Networks.

[20] Juan A, Faulin J, Jorba J, Riera D, Masip D, Barrios B (2010). On the use of Monte Carlo Simulation, Cache and Splitting Techniques to Improve the Clarke and Wright Savings Heuristics. J Oper Res Soc.

[21] Polastre J, Hill J, Culler D Versatile Low Power Media Access for Wireless Sensor Networks.

